# Supplementary material for: Experimental determination and mathematical modeling of standard shapes of forming autophagosomes
Source: Nat Commun. 2024 Jan 2;15:91. doi: 10.1038/s41467-023-44442-1 (PMC10762205; doi:10.1038/s41467-023-44442-1)
Supplement: Supplementary file 1 — Supplementary Information [file 41467_2023_44442_MOESM1_ESM.pdf]

Supplementary Information

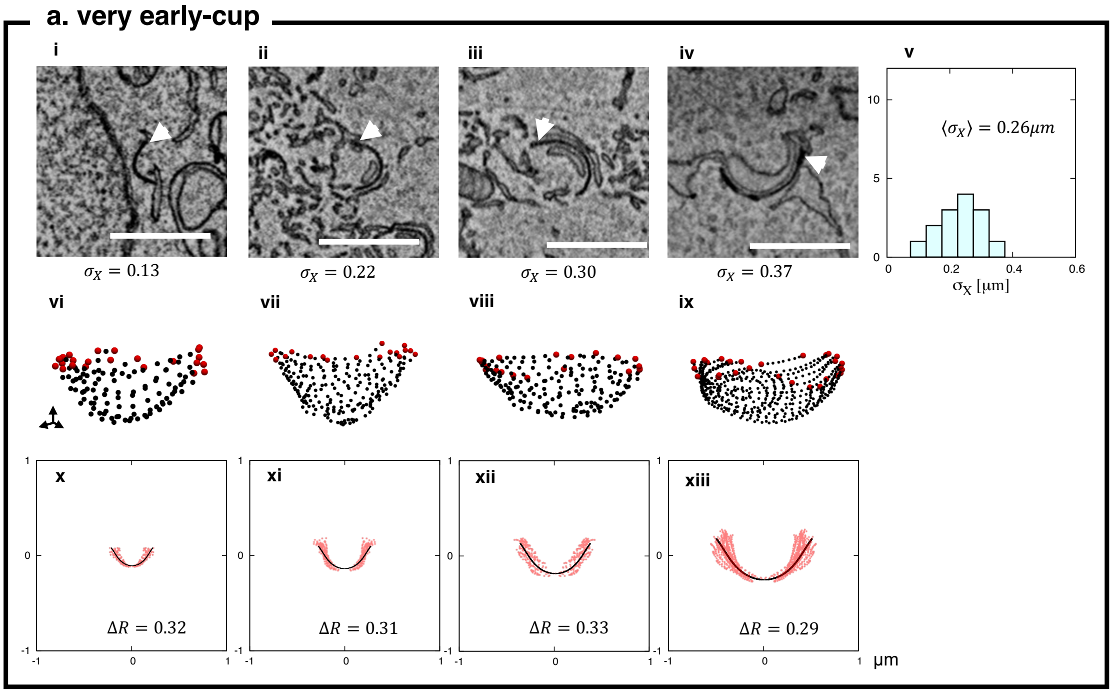

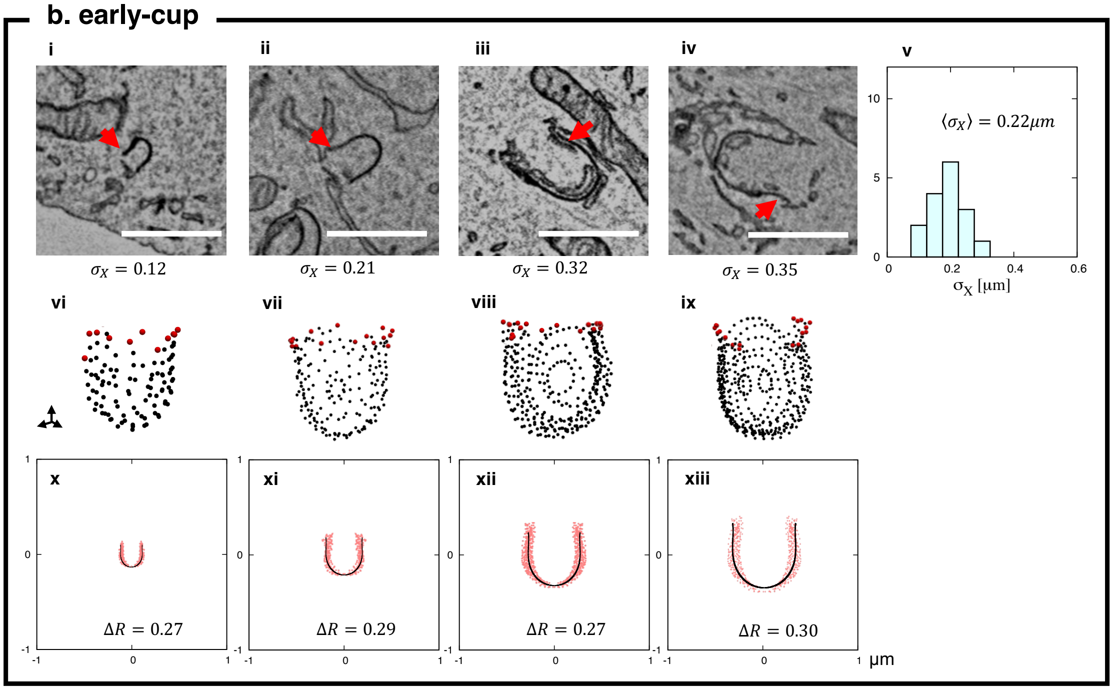

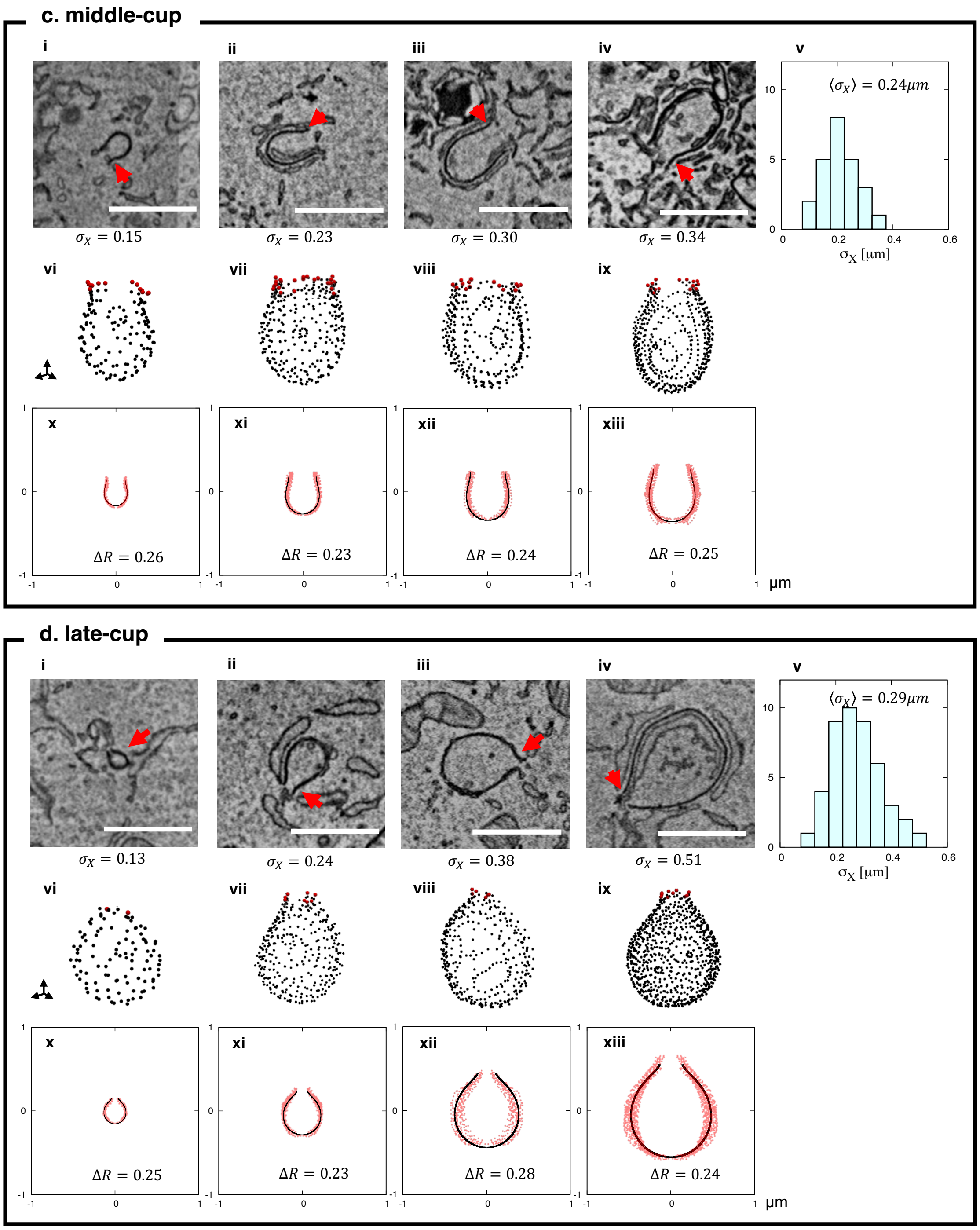

Supplementary Figure 1. Comparison of individual shapes of phagophores of different sizes obtained experimentally with the theoretical model.

(**i**–**iv**) Electron microscope images of phagophores of various sizes (σ_X) at the very early-cup (**a**), early-cup (**b**), middle-cup (**c**), and late-cup stages (**d**). The white arrows indicate the rims of very early-cup shaped phagophores and the red arrows indicate the catenoidal rims of early-, middle- and late-cup-shaped phagophores; scale bar, 1 μm. (**v**) Histogram

of the deviation of the point set, representing the size of the shape, for each shape. $\langle\sigma_X\rangle$ is the average value for each shape. (**vi**–**ix**) 3D reconstruction of point clouds of the phagophores extracted from serial EM images. (**x**–**xiii**) The theoretically calculated shapes (black lines) are superposed over the point clouds (red dots), where the unit of length is micrometers. The same theoretical shapes as shown in Fig. 6 were used. The relative errors between experimental and theoretical values ($\Delta R = \left|R_{exp} - R_{theo}\right|/R_{exp}$) for each shape are shown in each panel, where R is the norm in polar coordinates (see Fig. S9**a**). Source data are provided as a Source Data file.

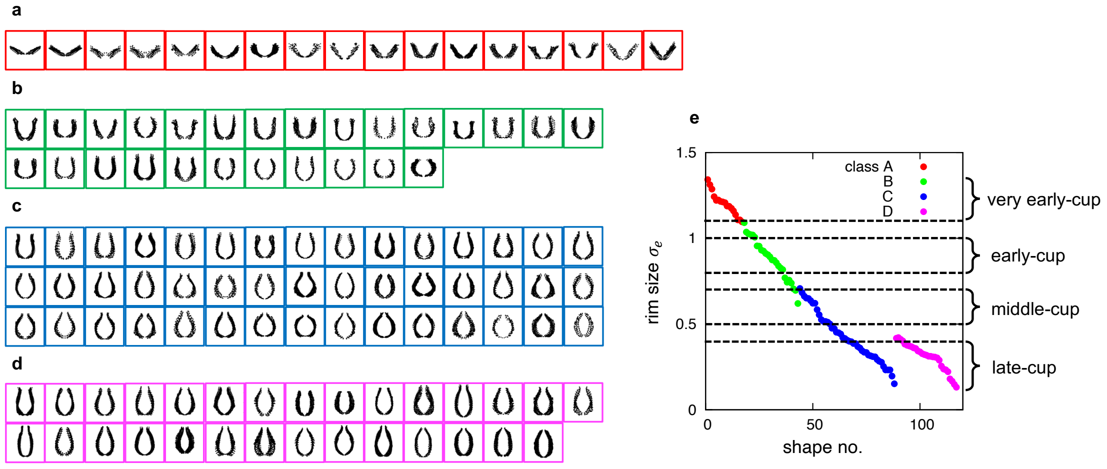

Supplementary Figure 2. Unsupervised machine learning clustering of phagophore shapes.

Morphological images of phagophores were classified into four categories using the k-means method of unsupervised clustering learning. (**a**–**d**) Point clouds representing the morphology of each phagophore projected onto a two-dimensional plane belonging to each category are shown. (**e**) The rim length of phagophores in each category. Classes **a**–**d** correspond to the shapes of point clouds shown in panels **a**–**d**. Source data are provided as a Source Data file.

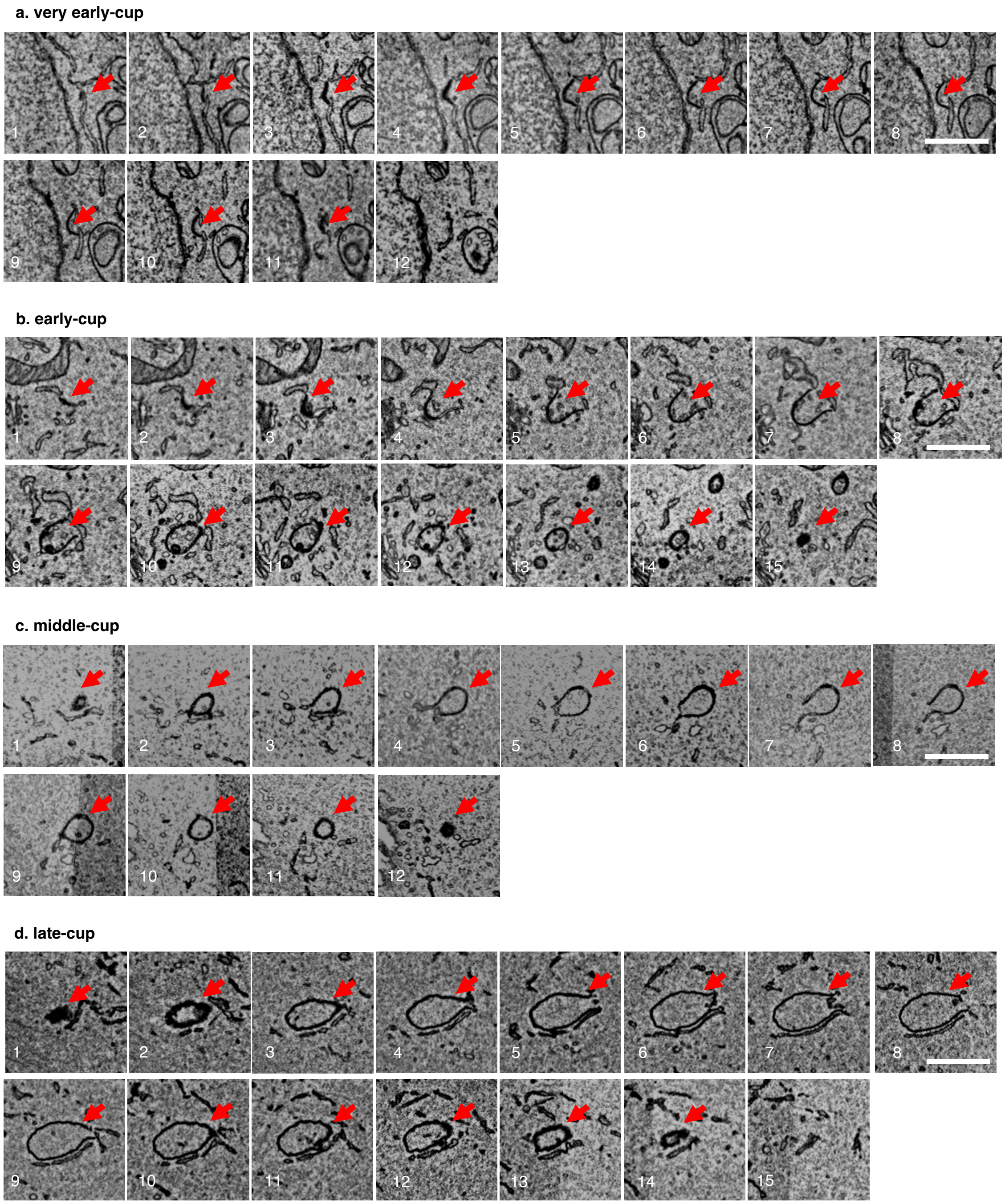

Supplementary Figure 3. Three-dimensional electron microscopy images of phagophores.

Examples of serial electron microscopy images of phagophores at the very early-cup (**a**), early-cup (**b**), middle-cup (**c**), and late-cup (**d**) stages. The red arrows indicate phagophores. The numbers represent the order of the serial images; scale bar, 1 μm.

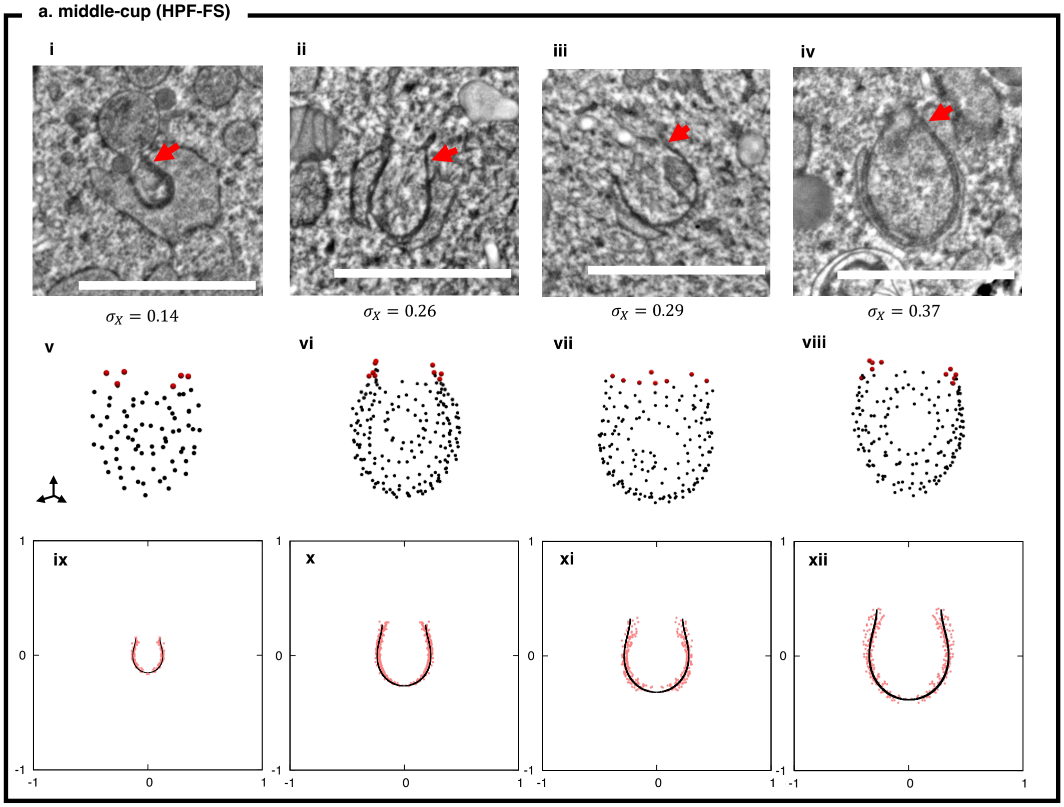
a. middle-cup (HPF-FS)
i
ii
iii
iv
σ_X = 0.14
σ_X = 0.26
σ_X = 0.29
σ_X = 0.37
v
vi
vii
viii
ix
x
xi
xii
1
0
-1
-1
0
1

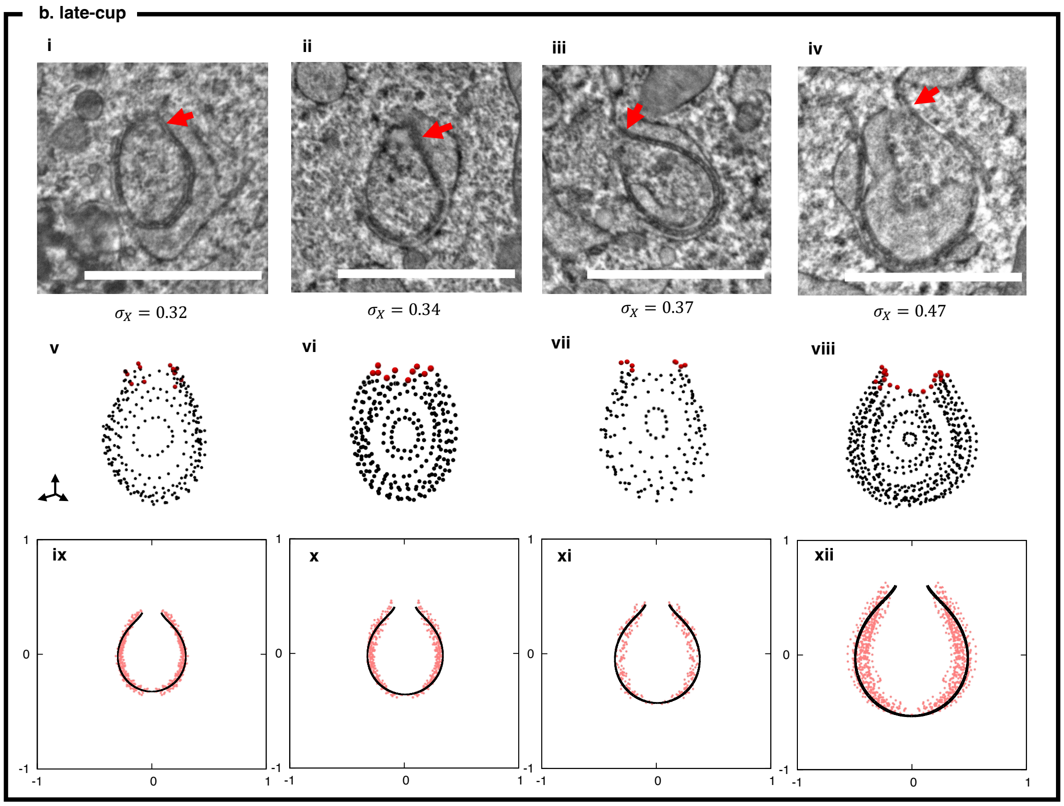
b. late-cup
i
ii
iii
iv
σ_X = 0.32
σ_X = 0.34
σ_X = 0.37
σ_X = 0.47
v
vi
vii
viii
ix
x
xi
xii
1
0
-1
-1
0
1

Supplementary Figure 4. High-pressure freezing and freeze substitution (HPF-FS) electron microscope images of cup-shaped phagophores.

(**i**–**iv**) HPF-FS electron microscopy images of phagophores of various sizes (σ_X) at the middle-cup (**a**) and late-cup stages (**b**). The red arrows indicate the catenoidal rims of phagophores; scale bar, 1 μm. (**v**–**viii**) 3D reconstruction of point clouds of the phagophores extracted from serial EM images. (**ix**–**xii**) The theoretically calculated shapes (black lines) are superposed over the point clouds (red dots), where the unit of length is micrometers. The same theoretical shapes as shown in Fig. 6 were used. Source data are provided as a Source Data file.

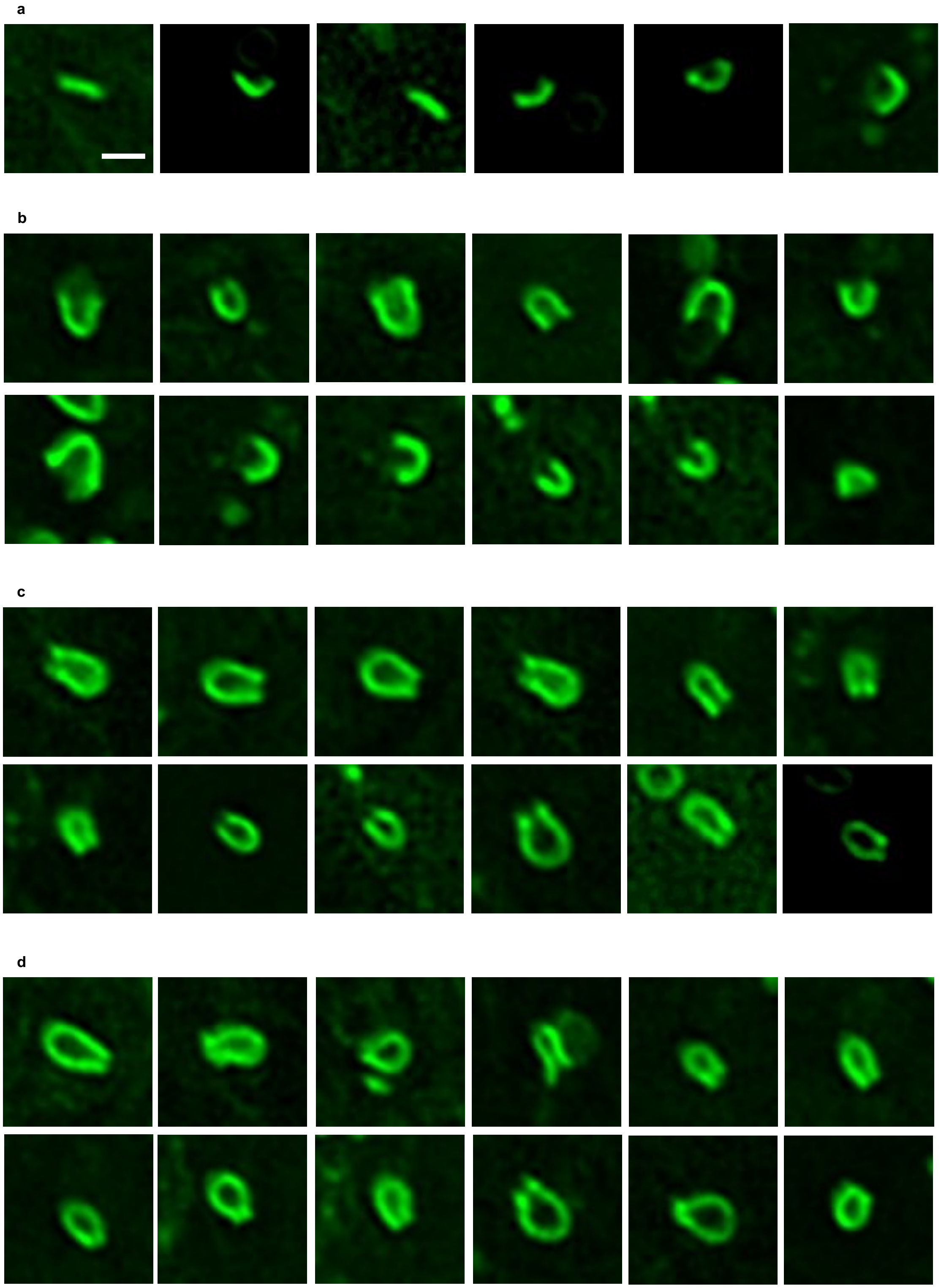

Supplementary Figure 5. In vivo fluorescent images of phagophores.

Live-cell imaging of mouse embryonic fibroblasts expressing GFP-LC3B under starvation conditions; scale bar, 1 μm. Snap-shot images of phagophores were classified into very early-cups (**a**), early-cups (**b**), middle-cups (**c**), and late-cups (**d**).

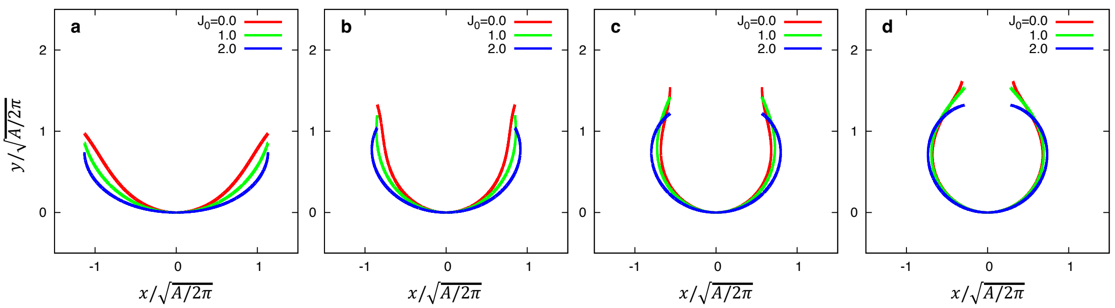

Supplementary Figure 6. Dependences of the shapes on spontaneous curvature.

Membrane shapes obtained based on the bending energy with Gaussian modulus $\kappa_G = -0.2\kappa_b$ and rim radii $l = 0.8$ (**a**), $l = 0.6$ (**b**), $l = 0.4$ (**c**), and $l = 0.2$ (**d**) for several values of spontaneous curvature J_0. The unit of length was non-dimensionalized by the length $\sqrt{A/2\pi}$. Source data are provided as a Source Data file.

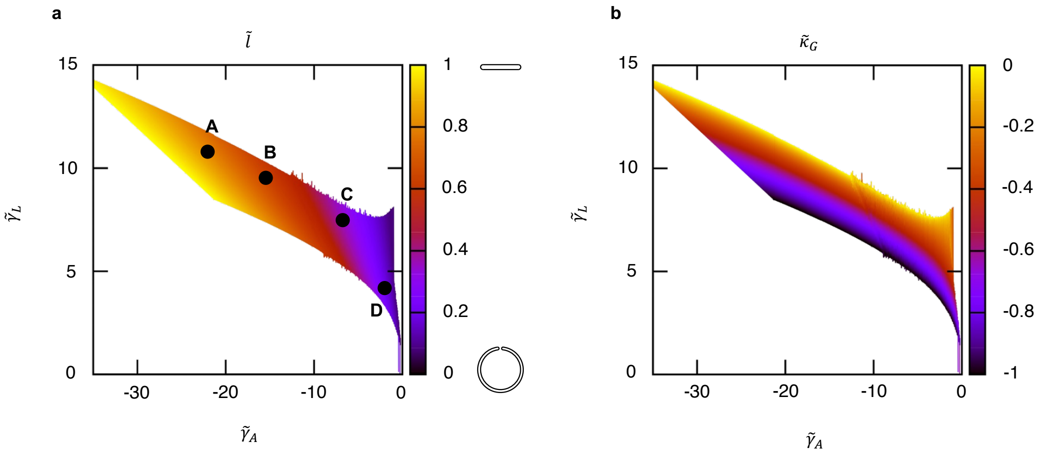

Supplementary Figure 7. Phase diagram of the membrane shapes on the membrane elastic moduli.

(**a**) Phase diagrams of the rim radius $\tilde{l}$ were calculated on the $\tilde{\gamma}_A$-$\tilde{\gamma}_L$ plane. The parameter sets corresponding to Figs. 6**a**–**d** are represented as black dots. (**b**) The Gaussian modulus $\tilde{\kappa}_G$ at the corresponding position in panel A. The parameters were nondimensionalized as $\tilde{l} = l/\sqrt{A}$, $\tilde{\kappa}_G = \kappa_G/\kappa_b$, $\tilde{\gamma}_A = \gamma_A A/\kappa_b$, and $\tilde{\gamma}_L = \gamma_L\sqrt{A}/\kappa_b$, respectively. The white areas indicate regions in which the membrane cannot be realized in simple geometry, for example, because the sign of x or dz changes. Source data are provided as a Source Data file.

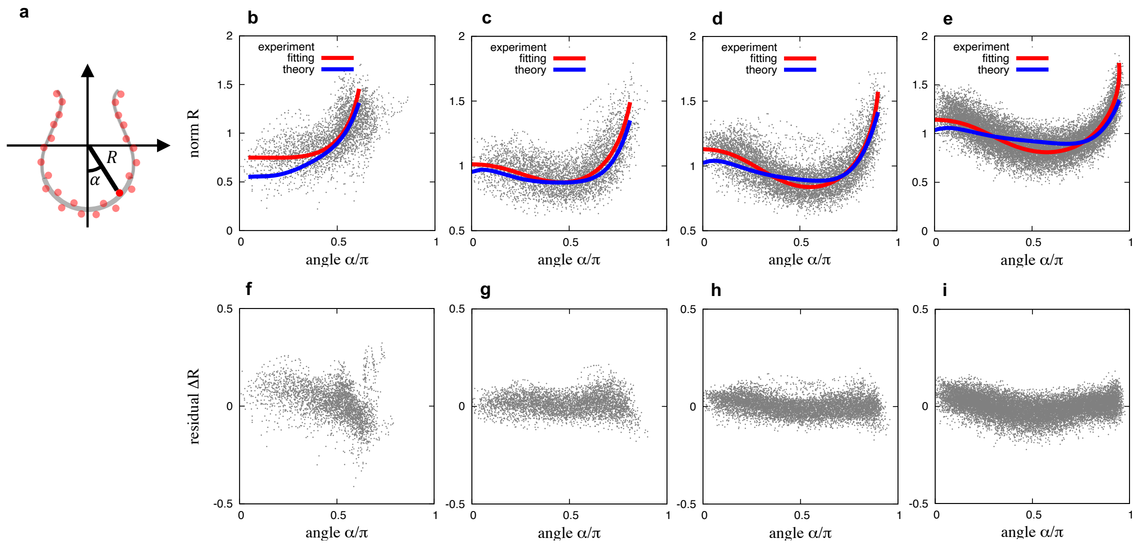

Supplementary Figure 8. Comparison between experimental and theoretical results.

(**a**) The norm R and angle α, as polar coordinates, are shown. (**b**–**e**) Angular dependence of the norm R in the polar coordinate representation of very early-cup (**b**), early-cup (**c**), middle-cup (**d**), and late-cup (**e**) shapes. The gray dots and the red lines represent the experimental point clouds (R_{exp}) and the fitting curves, respectively. The data are the same as those used in Fig. 4 (**a**–**d**). The blue lines represent the theoretical results (R_{theo}) in polar coordinates in Fig. 6. The length unit of the norm was the same as in Fig. 6. The relative errors between experimental and theoretical values ($\left|R_{exp} - R_{theo}\right|/R_{exp}$) for each shape were 29%, 20%, 20%, and 27%, respectively. (**f**–**i**) The norm residual ($R_{fit} - R_{theo}$) of the very early-cup (**f**), early-cup (**g**), middle-cup (**h**), and late-cup (**i**) shapes. Source data are provided as a Source Data file.

Supplementary Table 1. Parameters of the fitting function for point clouds obtained from electron microscopy (EM) images.

	a	b	c	d	χ_ν^2
very early-cup	1.92 (0.16)	1.88 (0.19)	1.61 (0.09)	0.89 (0.11)	0.041
early-cup	0.42 (0.02)	-0.49 (0.01)	0.06 (0.01)	0.91 (0.02)	0.018
middle-cup	0.307 (0.006)	-0.587 (0.005)	-0.263 (0.005)	0.840 (0.003)	0.015
late-cup	0.266 (0.003)	-0.464 (0.003)	-0.383 (0.004)	0.802 (0.002)	0.016

The parameter sets of the polynomial function [Eq. (5)] that fit the point cloud shown in Fig. 3**e**–**h**. The standard errors are in parentheses. The reduced chi-square (χ_ν^2), which represents the goodness of fit, is also shown. The shapes obtained from each polynomial function are shown in Fig. 4**a**–**d**.
