# Supplementary material for: Experimental determination and mathematical modeling of standard shapes of forming autophagosomes
Source: Nat Commun. 2024 Jan 2;15:91. doi: 10.1038/s41467-023-44442-1 (PMC10762205; doi:10.1038/s41467-023-44442-1)
Supplement: Supplementary file 3 — Description of Additional Supplementary Files [file 41467_2023_44442_MOESM3_ESM.pdf]

Description of Additional Supplementary Files

File name: Supplementary Movie 1
Description: Example of a rotating phagophore structure, corresponding to Fig. 2d.

File name: Supplementary Movie 2
Description: Rotating view of the superposed very early-cup morphology of a phagophore, corresponding to Fig. 3a.

File name: Supplementary Movie 3
Description: Rotating view of the superposed early-cup morphology of a phagophore, corresponding to Fig. 3b.

File name: Supplementary Movie 4
Description: Rotating view of the superposed middle-cup morphology of a phagophore, corresponding to Fig. 3c.

File name: Supplementary Movie 5
Description: Rotating view of the superposed late-cup morphology of phagophore, corresponding to Fig. 3d.
