# Supplementary material for: Experimental determination and mathematical modeling of standard shapes of forming autophagosomes
Source: Nat Commun. 2024 Jan 2;15:91. doi: 10.1038/s41467-023-44442-1 (PMC10762205; doi:10.1038/s41467-023-44442-1)
Supplement: Supplementary file 9 — Reporting Summary [file 41467_2023_44442_MOESM9_ESM.pdf]

nature portfolio

Corresponding author(s): Yuji Sakai and Noboru Mizushima

Last updated by author(s): Dec 6, 2023

Reporting Summary

Nature Portfolio wishes to improve the reproducibility of the work that we publish. This form provides structure for consistency and transparency in reporting. For further information on Nature Portfolio policies, see our Editorial Policies and the Editorial Policy Checklist.

Statistics

For all statistical analyses, confirm that the following items are present in the figure legend, table legend, main text, or Methods section.

n/a	Confirmed	
☐	☒	The exact sample size (*n*) for each experimental group/condition, given as a discrete number and unit of measurement
☐	☒	A statement on whether measurements were taken from distinct samples or whether the same sample was measured repeatedly
☒	☐	The statistical test(s) used AND whether they are one- or two-sided<br>*Only common tests should be described solely by name; describe more complex techniques in the Methods section.*
☒	☐	A description of all covariates tested
☒	☐	A description of any assumptions or corrections, such as tests of normality and adjustment for multiple comparisons
☐	☒	A full description of the statistical parameters including central tendency (e.g. means) or other basic estimates (e.g. regression coefficient) AND variation (e.g. standard deviation) or associated estimates of uncertainty (e.g. confidence intervals)
☒	☐	For null hypothesis testing, the test statistic (e.g. *F*, *t*, *r*) with confidence intervals, effect sizes, degrees of freedom and *P* value noted<br>*Give P values as exact values whenever suitable.*
☒	☐	For Bayesian analysis, information on the choice of priors and Markov chain Monte Carlo settings
☒	☐	For hierarchical and complex designs, identification of the appropriate level for tests and full reporting of outcomes
☒	☐	Estimates of effect sizes (e.g. Cohen's *d*, Pearson's *r*), indicating how they were calculated

Our web collection on statistics for biologists contains articles on many of the points above.

Software and code

Policy information about availability of computer code

Data collection	Electron microscope images: PC-SEM (ver. 7.1.0.10, JEOL), Array Tomography Supporter (ver. 1.2.5.0, System in Frontier Inc.)<br>Fluorescent microscope images: cellSens (ver. 2.4.1.198, Olympus)
Data analysis	Alignment of electron microscope images: StackerNEO (ver. 3.3.4.0, System in Frontier Inc.)<br>Extraction of membrane coordinates: Plot Digitizer ver. 2.6.8 (https://plotdigitizer.sourceforge.net/)<br>Numerical analyses: Fortran Intel(R) 64, ver. 2021.7.1<br>Custom code are available from https://github.com/YSakaiGH/autophagosome_shapes<br>Image processing: Fiji (Image J 1.54f)

For manuscripts utilizing custom algorithms or software that are central to the research but not yet described in published literature, software must be made available to editors and reviewers. We strongly encourage code deposition in a community repository (e.g. GitHub). See the Nature Portfolio guidelines for submitting code & software for further information.

Data

Policy information about availability of data

All manuscripts must include a data availability statement. This statement should provide the following information, where applicable:

- Accession codes, unique identifiers, or web links for publicly available datasets
- A description of any restrictions on data availability
- For clinical datasets or third party data, please ensure that the statement adheres to our policy

Source data for all graphs of all figures are provided with the paper as Supplementary Information. Source data are provided with this paper.

Research involving human participants, their data, or biological material

Policy information about studies with human participants or human data. See also policy information about sex, gender (identity/presentation), and sexual orientation and race, ethnicity and racism.

Reporting on sex and gender	N/A
Reporting on race, ethnicity, or other socially relevant groupings	N/A
Population characteristics	N/A
Recruitment	N/A
Ethics oversight	N/A

Note that full information on the approval of the study protocol must also be provided in the manuscript.

Field-specific reporting

Please select the one below that is the best fit for your research. If you are not sure, read the appropriate sections before making your selection.

☒ Life sciences ☐ Behavioural & social sciences ☐ Ecological, evolutionary & environmental sciences

For a reference copy of the document with all sections, see nature.com/documents/nr-reporting-summary-flat.pdf

Life sciences study design

All studies must disclose on these points even when the disclosure is negative.

Sample size	No statistical methods were used to predetermine sample size. Sample sizes were determined considering factors such as the desired level of significance, effect size, and variability within the population.
Data exclusions	No data were excluded from any of the experimental studies presented in the manuscript.
Replication	For the electron microscopy experiments, images were obtained from serial ultrathin sections from two independent resin-embedded blocks, and for the fluorescence microscopy experiments, two biological replicates were performed, each with consistent results.
Randomization	For electron microscopy experiments, serial section images of 117 autophagosomes were randomly selected for analysis from two resin-embedded blocks (6 and 17 cells from each block). For fluorescence microscopy experiments, more than 30 cup-shaped autophagosomes from two biological replicates were observed by 3D time-lapse microscopy.
Blinding	Blinding was not possible and not necessary in this study, since there is no data of distinct groups that could be analyzed blinded.

Reporting for specific materials, systems and methods

We require information from authors about some types of materials, experimental systems and methods used in many studies. Here, indicate whether each material, system or method listed is relevant to your study. If you are not sure if a list item applies to your research, read the appropriate section before selecting a response.

Materials & experimental systems

n/a	Involved in the study	
☒	☐	Antibodies
☐	☒	Eukaryotic cell lines
☒	☐	Palaeontology and archaeology
☒	☐	Animals and other organisms
☒	☐	Clinical data
☒	☐	Dual use research of concern
☒	☐	Plants

Methods

n/a	Involved in the study	
☒	☐	ChIP-seq
☒	☐	Flow cytometry
☒	☐	MRI-based neuroimaging

Eukaryotic cell lines

Policy information about cell lines and Sex and Gender in Research

Cell line source(s)	Mouse embryonic fibroblasts established from C57BL/6 mice.
Authentication	N/A
Mycoplasma contamination	Cells were confirmed to be negative for mycoplasma contamination by observation by fluorescence microscopy.
Commonly misidentified lines (See ICLAC register)	N/A

Plants

Seed stocks	N/A
Novel plant genotypes	N/A
Authentication	N/A
